# Protective effects of hydrogen rich saline solution in rats with experimental myocardial ischemia reperfusion injury

**DOI:** 10.1016/j.heliyon.2023.e22973

**Published:** 2023-11-28

**Authors:** Zeynep Köksal, Ömer Kurtipek, Mustafa Arslan, Ali Doğan Dursun, Zeynep Yığman, Abdullah Özer

**Affiliations:** a Haymana State Hospital Department of Anesthesiology and Reanimation, Ankara, Turkey; b Gazi University Faculty of Medicine Department of Anesthesiology and Reanimation, Ankara, Turkey; c Gazi University, Life Sciences Application and Research Center, Ankara, Turkey; d Gazi University, Laboratory Animal Breeding and Experimental Researches Center (GÜDAM), Ankara, Turkey; e Atılım University Faculty of Medicine Department of Physiology, Ankara, Turkey; f Gazi University Faculty of Medicine, Department of Histology and Embryology, Ankara, Turkey; g Gazi University Neuroscience and Neurotechnology Center of Excellence (NÖROM), Ankara, Turkey; h Gazi University Faculty of Medicine, Department of Cardiovascular Surgery, Ankara, Turkey

**Keywords:** Hydrogen-rich saline solution, Ischemia reperfusion, Myocard, Total antioxidant status, Total oxidative status

## Abstract

**Aim:**

The aim of our study is to show whether the administration of hydrogen-rich saline solution (HRSS) intraperitoneally before left main coronary artery (LAD) ischemia protects the myocardium against ischemia-reperfusion (IR) injury.

**Materials and methods:**

After ethics committee approval, 24 Wistar Albino rats were divided into 4 groups, 6 rats in each group. For experimental IR, myocardial ischemia was performed by LAD ligation. Left thoracotomy was performed without ischemia in the Control group (Group C). Left thoracotomy was performed without myocardial ischemia to the rats in the HRSS group, and HRSS was given intraperitoneally (ip) at a rate of 10 ml/kg throughout the procedure. In the MIR-HRSS group, a single dose of 10 ml/kg HRSS was administered 5 min before reperfusion. Histopathological and biochemical parameters were compared in myocardial tissue samples taken at the end of the reperfusion period.

**Results:**

When the groups were compared among themselves in terms of TOS and TAS levels, there was a significant difference between the groups (p = 0.006, p = 0.002). The severity of cardiomyocyte degeneration was significantly greater in MIR group than that in the control and HRSS groups (p = 0.002 and p = 0.001, respectively), as well as severity score of cardiomyocyte degeneration was higher in MIR-HRSS group compared with HRSS group (p = 0.035).

**Conclusion:**

Our study shows that HRSS is protective in IR injury, with the application of HRSS 5 min before reperfusion, interstitial edema severity, subendocardial haemorrhage are reduced, and oxidant status parameters are increased, while antioxidant status parameters are decreased. We believe that when it is supported by other studies, the protective effects of HRSS on IR damage will be shown in detail and its indications will be expanded.

## 1 Introduction

Ischemic heart disease remains current as one of the most important causes of morbidity and mortality. About half of the deaths that occur each year in developed countries are due to problems with the cardiovascular system. The most common form is called “coronary atherosclerotic heart disease”, and the coronary blood flow feeding the myocardium due to atherosclerotic plaques and vasospasm is reduced enough to give clinical and pathological symptoms [1].

Myocardial ischemic injury results from severe disruption of coronary blood flow and produces a range of clinical syndromes. As a result of decades of intensive research, a detailed understanding of the complexity of the myocardium's response to the ischemic state is now available. Myocardial ischemia results in a characteristic pattern of metabolic and structural changes that cause irreversible damage [2]. Ischemia, which is defined as oxygen deprivation of these tissues or organs as a result of insufficient perfusion of organs and tissues due to decreased arterial or venous blood flow, leads to cell death as a result of depletion of cellular energy stores and accumulation of toxic metabolites. Ischemic tissue requires reflow of blood for both cell regeneration and clearance of toxic metabolites. However, reperfusion of ischemic tissue paradoxically causes much more serious damage to the tissue than ischemia alone [3].

During the ischemic period, metabolic and structural changes occur in the cell. With the cessation of blood flow to the tissue, cellular oxidative phosphorylation decreases and synthesis of high-energy phosphates such as adenosine 5′-triphosphate (ATP) and phosphocreatine decreases. With the discharge of energy stores in the cell, the Na + K + -ATP less pump in the cell membrane is inhibited. As a result, intracellular Na+ and Ca2+ ion concentrations increase. An increase in Ca2+ ion concentration in the cell is cytotoxic to the cell. As a matter of fact, in this period, with the change of ion concentration in the cell, there is an increase in the production of proinflammatory cytokines and leukocyte adhesion molecules, while there is a decrease in the formation of antioxidant enzymes. This makes, causing the cell vulnerable to damage during the reperfusion period [4]. Reperfusion after ischemia activates many transcription factors such as inflammatory response-related intracellular adhesion molecule (ICAM), interleukin 1-beta (IL-1β), IL-8, tumor necrosis factor alpha (TNF-α). If blood flow is restored after reperfusion, substances formed as a result of oxidation of metabolites spread throughout the body through the systemic circulation. Free oxygen radicals (FOR) are the most important toxic substances formed as a result of the presentation of oxygen to the ischemic area. Under normal conditions, antioxidant mechanisms remove the formed FOR from the environment and it is in equilibrium in the organism. If the antioxidant systems are insufficient, the amount of FOR increases and severe reperfusion injury occurs [5].

In addition, activated adhesion molecules and cytokines can initiate a systemic inflammatory response. These responses are defined as ischemia-reperfusion (IR) injury [6]. Various methods have been developed to prevent IR injury. Ohsawa et al. [7] found that hydrogen (H2) selectively reduces the hydroxyl radical, which is the most cytotoxic of reactive oxygen species (ROS), and protects cells effectively; showed that it does not interact with other ROSs of physiological importance. In addition, inhalation of H2 gas has been shown to reduce infarct size in a rat model of myocardial IR (MIR) injury [8].

As in other therapeutic modalities, effects of molecular hydrogen have been tested mostly on rodents but have also been studied in six human diseases. The reported human diseases include diabetes mellitus type II [9], metabolic syndrome [10], hemodialysis [11], inflammatory and mitochondrial myopathies [12], brain stem infarction [13], and radiation-induced adverse effects for liver tumor [14].

The aim of this study is to show whether intraperitoneal administration of hydrogen-rich saline solution (HRSS) before left main coronary artery ischemia protects myocardium against IR injury.

## 2 Materials and methods

### 2.1 Animals and experimental protocol

The study was conducted in the GUDAM Laboratory of Gazi University with the consent of Experimental Animals Ethics Committee of Gazi University (G.Ü.ET-20-057). All of the procedures were performed according to the accepted standards of the Guide for the Care and Use of Laboratory Animals. 24 male Wistar Albino rats (250–275 g) were used. We chose male rats because we had an appropriate number of male rats in our laboratory. By selecting all rats of the same sex, we minimized the differences that may occur due to gender.

The rats were kept at 20–21 °C in cycles of 12 h of daylight and 12 h of darkness and had free access to food until 2 h before the anesthetic procedure. The animals were randomly separated into four groups, each containing 6 rats. Control group (C), HRSS group (HRSS), Myocardial ischemia-reperfusion (MIR), and Myocardial ischemia-reperfusion + HRSS (MIR-HRSS).

Rats underwent left thoracotomy and received HRSS, 10 ml/kg, administrated via i.p. route 30 min before ligating the LAD [15]. A small plastic snare was threaded through the ligature and placed in contact with the heart. The artery could then be occluded by applying tension to the ligature (30 min), and reperfusion was achieved by releasing the tension (120 min). However, after the above procedure, the coronary artery was not occluded or reperfused in the control, and HRSS rats. Rats were anesthetized with 50 mg/kg (i.p.) ketamine and 10 mg/kg (i.p.) xylazine. We optimized the conditions for the effect of ketamine on cardiac output by administering the same amount of ketamine to all rats, including the control group.

The trachea was cannulated for artificial respiration. The chest was shaved and each animal was fixed in a supine position on the operating table. The chest was opened by left thoracotomy followed by sectioning the fourth and fifth ribs about 2 mm to the left of the sternum. Positive-pressure artificial respiration was started immediately with room air, using a volume of 1.5 ml/100 g body weight at a rate of 60 strokes/min. Sodium heparin (500 IU/kg) was administered through the peripheral vein in the tail. After the pericardium was incised, the heart was exteriorized with gentle pressure on the right side of the rib cage. An 8/0 silk suture attached to a 10-mm micropoint reverse-cutting needle was quickly placed under the left main coronary artery. The heart was then carefully replaced in the chest and the animal was allowed to recover for 20 min (MIR, and MIR-HRSS groups). During reperfusion, the thorax was covered with a moist sterile pad.

Anesthesia was maintained in the with injections of 20 mg/kg ketamine if a positive reaction to surgical stress or intermittent tail pinch was observed. Following the end of the reperfusion period, all rats were anesthetized with ketamine (50 mg/kg) and sacrificed by collecting blood (5–10 ml) from intracardiac.

After 120 min of reperfusion, the myocardial tissue of the rats was removed under anesthesia, myocardial tissue specimens were excised for subsequent biochemical and histopathological analysis. Myocardial tissues were removed in such a way that their integrity was not impaired and they were not traumatized. It was placed in 10 % formol for histopathological examination, frozen in liquid nitrogen and stored at −80 °C for biochemical examination.

### 2.2 Histopathological analysis

For histological analysis cardiac tissue samples were fixed in 10 % neutral buffered formalin for 48 h. Fixed samples were dehidrated through grading alcohol series (70 %, 80 %, 96 %, %96, 100 %, 100 %), cleared in xylene and embedded in paraffin. Then 5 μm thick sections parallel to apex-basis plane were cut from paraffin blocks by using a microtome (Leica RM2245, Germany) and stained with hematoxylin (05–06004/L, BioOptica, Italy) and eosin (05–11007/L, BioOptica, Italy). Hematoxylin and eosin (H&E) stained sections were examined under 200 × and 400 × magnifications by using a light microscope (Leica DM 4000B, Germany) equipped with a computer and images were taken by using the software Leica LAS V4.9 (Germany). Cardiac ischemia-reperfusion injury, in regards to histological changes including interstitial edema, cardiomyocyte degeneration (i.e., swelling, vacuolization), myocardial hypercontraction bands and subendocardial haemorrhage, was evaluated and a modified scoring system based on previous studies was applied for this evaluation [15,16]. Accordingly, aforementioned lesions indicating myocardial injury were scored individually in respect of severity (0 = none, 1 = mild, 2 = moderate, and 3 = severe) and distribution (0 = none, 1 = focal, 2 = multifocal, and 3 = diffuse). Mean scores of severity and distribution of each variable were determined for each heart and then for each group and compared between the groups.

### 2.3 Homogenization of tissues

The heart tissue was collected into a sterile eppendorf tube and kept at −80 °C until being analyzed for total antioxidant/oxidant status, paraoxanase and arylesterase activities. Without being allowed to dissolve, the tissues were quickly weighed on a precision scale and separated as 80–100 mg using a no. 22 lancet (PLUSMED®). Tissue fragments were smashed in the presence of liquid nitrogen in a porcelain bowl. The powdered tissue was transferred to the homogenization tube (099C S3, Glas-Col) and for each gram of tissue, 140 mM KCI solution was added such that the dilution was 1/10 (w/v). In order not to increase the temperature, the homogenization tube was held in a snow-filled glass beaker and after homogenization with a homogenizer (Glas_Col K5424®) at a speed of 50 revolutions per minute (rpm) for 2 min (pestle, 099C S21G, Glas-Col), the homogenization process was completed. The homogenate was transferred to an eppendorf tube. Eppendorf tubes were coated with parafilm and then centrifuged (Hettich Micro 200R®) for 10 min at 3000 rpm. After centrifugation, the supernatant was taken into another eppendorf tube and TOS, TAS paraoxanase and arylesterase activity levels were measured.

### 2.4 Total antioxidant status (TAS)

TAS was measured by a TAS test kit (RelAssay Diagnostic®, Turkey). For TAS measurement, as described in kit's procedure, 500 μL of reagent 1 (measurement buffer) and 30 μL of sample were mixed and absorbance was measured at 660 nm by a spectrophotometer (NanoDrop® ND-1000, Thermo Scientific) (A1). A volume of 75 μL of reagent 2 (colored 2,2-azino-bis-3-ethylbenzothiazoline-6-sulfonic acid; ABTS) was added to the mixture in the Eppendorf tube. The tube was coated with paraffin and incubated for 5 min in a hot water bath at 37 °C (ST 30, NUVE). After incubation, absorbance measurement was performed at 660 nm (A2). For standard measurement, the Trolox Eq solution at a concentration of 1 mmol/L was used instead of the sample. The fi rst and second measurements were made for three times and their averages were measured. The absorbance change (ΔAbs) was calculated by subtracting the first absorbance value (A1) from the second absorbance value (A2). TAS levels were calculated using the formula given in the kit and expressed as mmol Trolox Eq/L.

TAS = [(ΔAbsH2O - ΔAbs Sample) / (ΔAbs H2O - ΔAbs Standard)]

### 2.5 Total oxidative status (TOS)

TOS was measured by a TOS test kit (RelAssay Diagnostic®, Turkey). For TOS measurement, as described in kit's procedure, 500 μL of reagent 1 (measurement buffer) and 75 μL of sample were mixed and absorbance was measured at 530 nm by a spectrophotometer (NanoDrop® ND-1000, Thermo Scientific; A1). A volume of 25 ml of reagent 2 (Pro-chromogenic solution) was added to the mixture. The tube was coated with paraffin and incubated for 5 min in a hot water bath at 37 °C (ST 30, NUVE). After incubation, absorbance measurement was performed at 530 nm (A2). A standard solution containing 10 μmol/L hydrogen peroxide (H_2_O_2_) equivalent/liter given in the kit was used for standard measurement. The first and second measurements were made for three times and their averages were measured. The absorbance change (ΔAbs) was calculated by subtracting the first absorbance value (A1) from the second absorbance value (A2). TOS levels were calculated using the formula given in the kit and expressed as mmol H_2_ O_2_ Eq/L.

TOS = [(ΔAbs sample)/(ΔAbs standard)] x Standard Concentration (10 μmol/L).

### 2.6 Paraoxanase (PON) measurement/arylesterase measurement

Paraoxonase activities were measured spectrophotometrically using commercially available kits (RelAssay Diagnostic®, Turkey).

The rate of paraoxon hydrolysis (diethylpnitrophenylphosphate in 50 mM glycine/NaOH, pH 10.5 containing 1 mM CaCl2) was measured by monitoring the increase of absorption at 412 nm at 37 °C. The amount of generated *p*-nitrophenol was calculated from the molar absorption coefficient at pH 8.5, which was 18.290 M−1 cm−1 at pH 10.5. One enzyme unit was defined as the amount of enzyme that catalyzed the hydrolysis of 1 μmol of substrate at 37 °C (U/L).

Phenylacetate was used as a substrate to measure the arylesterase activity. Phenylacetate when hydrolysed, produces phenol and aceticacid. Enzymatic activity was calculated from the molar absorption coefficient of the produced phenol, 4000 M−1 cm−1 (Primary wavelength: 548 nm, secondary wavelength 700 nm). One unit of arylesterase activity was defined as 1 μmol phenol generated per minute at 37 °C, and expressed as U/L (Relassay, Turkey).

### 2.7 Statistical analysis

All the data were processed by variance analysis in the Statistical Package for the Social Sciences (SPSS, Chicago, IL, USA)22.0 program for Windows statistical software. The Shapiro–Wilk test and Q–Q plot test were used to assess data distribution. Biochemical and histopathological parameters were tested using the Kruskal-Wallis test. Bonferroni-adjusted Mann–Whitney *U* test was used after significant Kruskal–Wallis to determine which group differs from the other. The data were expressed as mean ± standard error (Mean ± SE). Value of p < 0.05 was considered statistically significant.

## 3 Results

### 3.1 Results of histopathological analysis

**Fig. 1:**
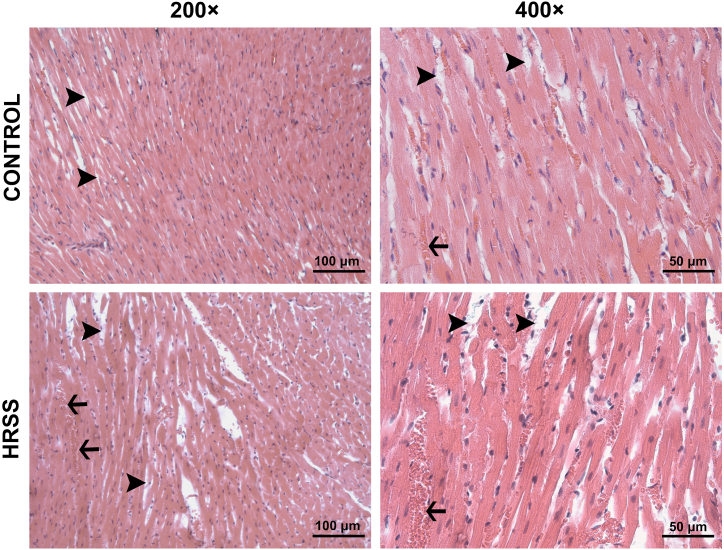
H&E stained representative micrographs of myocardial tissue sections from control and HRSS groups. A mild interstitial edema (**arrowhead**) and congestion (**arrow**) were present in the sections of hearts from both the control and HRSS groups. CONTROL, control group; HRSS, hydrogen-rich saline solution group. H&E, hematoxylin and eosin stain; 200 × and 400 × magnification.

**Fig. 2:**
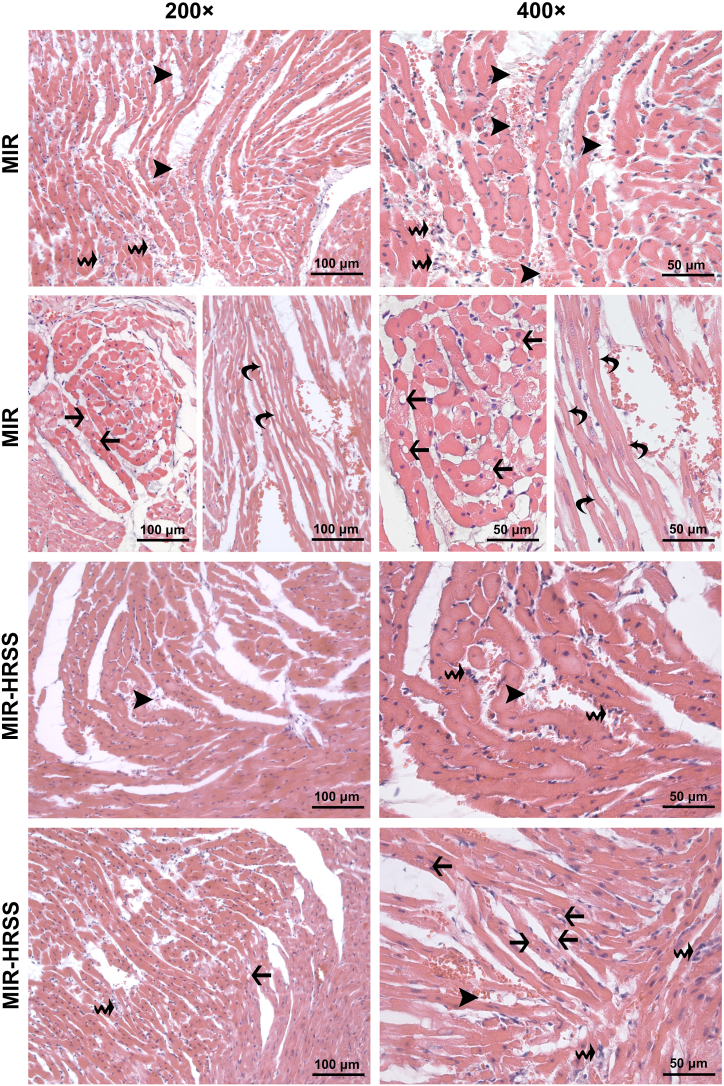
H&E stained representative micrographs of myocardial tissue sections from MIR and MIR-HRSS groups. When compared to the control and HRSS groups, more prominent interstitial edema (**arrowhead**) accompanied by erythrocyte extravasation in most regions, and mild leukocyte infiltration (**waved arrow**) were noted in the MIR and MIR-HRSS groups. In the sections of hearts from MIR and MIR-HRSS groups, damaged cardiomyocytes displaying degenerative changes varying from hydropic changes such as edema and small vesicles to large cytoplasmic vacuoles (**arrow**), were observed. Furthermore, in the sections of hearts from MIR group, wavy fibers (necrotic cardiomyocytes with no cross striations and nucleus) (**curved arrow**), were noticed in the areas with marked interstitial edema. MIR, myocardial ischemia-reperfusion group; MIR-HRSS, myocardial ischemia-reperfusion + hydrogen-rich saline solution group. H&E, hematoxylin and eosin stain; 200 × and 400 × magnification.

**Fig. 3:**
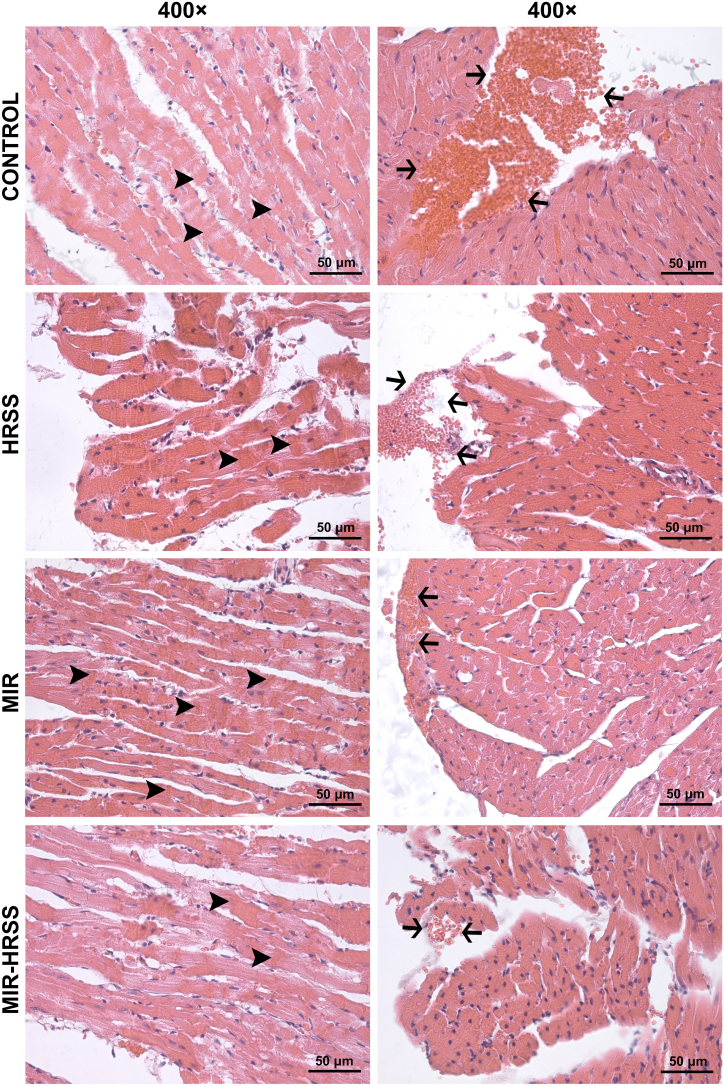
H&E stained representative micrographs of myocardial tissue specimens of animals from control, HRSS, MIR and MIR-HRSS groups. Myocardial hypercontraction bands (**arrowhead**) and subendocardial haemorrhage (**arrow**) were found to be similar in all groups. CONTROL, control group; HRSS, hydrogen-rich saline solution group; MIR, myocardial ischemia-reperfusion group; MIR-HRSS, myocardial ischemia-reperfusion + hydrogen-rich saline solution group. H&E, hematoxylin and eosin stain; 400 × magnification.

Interstitial edema, one of the parameters used to evaluate tissue injury, was similarly slight in the control and HRSS groups (Fig. 1), while more prominent interstitial edema accompanied by erythrocyte extravasation and a mild leukocyte infiltration was observed in MIR and MIR-HRSS groups (Fig. 2). The severity level of interstitial edema was found to be significantly different between the groups (p = 0.003). In this regard, severity score of interstitial edema was significantly higher in MIR group compared to the control and HRSS groups (p = 0.001 and p = 0.001, respectively), whereas it was lower in MIR-HRSS group compared to MIR group (p = 0.007). Distribution of interstitial edema was also significantly different between the groups (p = 0.046). Distribution of interstitial edema was comparable in all of the control, HRSS and MIR-HRSS groups (p = 0.034), while it was more widely distributed in MIR group compared to the control group (p = 0.011) (Table 1). Considering microscopic changes indicating cardiomyocyte degeneration such as swelling and vacuolization, the severity of lesions was found significantly different between the groups (p = 0.002). The severity of cardiomyocyte degeneration was significantly greater in MIR group than that in the control and HRSS groups (p = 0.002 and p = 0.001, respectively), as well as severity score of cardiomyocyte degeneration was higher in MIR-HRSS group compared to HRSS group (p = 0.035) (Fig. 1, Fig. 2). Also, distribution of cardiomyocyte degeneration was significantly different between the groups (p = 0.017), that the lesions were more widespread in MIR group than in the control and HRSS groups (p = 0.006 and p = 0.006, respectively), (Table 1). Myocardial hypercontraction bands, another histopathological parameter to evaluate the cardiac ischemia-reperfusion injury, was observed more specifically in MIR group and least in HRSS group, however differences between groups were not significant, and both the severity and distribution scores were similar in all groups (p = 0.121 and p = 0.246, respectively) (Table 1; Fig. 3). There was not significant difference between the groups in respect of the severity and distribution of subendocardial haemorrhage, as well (p = 0.404 and p = 0.178, respectively), (Table 1; Fig. 3).

**Table 1:** Comparison of histopathological evaluation of myocardial tissue [Mean ± SE].

	Group C (n = 6)	Group HRSS (n = 6)	Group MIR (n = 6)	Group MIR-HRSS (n = 6)	P**
Severity of interstitial edema	0.83 ± 0.17*	0.83 ± 0.17*	1.67 ± 0.21	1.00 ± 0.00*	0.003
Distribution of interstitial edema	0.80 ± 0.20*	1.33 ± 0.33	2.00 ± 0.37	1.66 ± 0.21	0.046
Severity of myofiber degeneration (i.e., myofiber swelling, vacuolization)	0.67 ± 0.21*	0.50 ± 0.22*	2.00 ± 0.37	1.33 ± 0.21&	0.002
Distribution of myofibre degeneration (i.e., myofiber swelling, vacuolization)	0.60 ± 0.25*	0.67 ± 0.33*	1.83 ± 0.31	1.16 ± 0.17	0.017
Severity of subendocardial haemorrhage	0.50 ± 0.22	0.50 ± 0.22	0.67 ± 0.22	0.17 ± 0.17	0.404
Distribution of subendocardial haemorrhage	0.50 ± 0.22	0.50 ± 0.22	1.00 ± 0.37	0.17 ± 0.17	0.178
Severity of myocardial hypercontraction bands	0.33 ± 0.21	0.17 ± 0.17	1.00 ± 0.37	0.67 ± 0.21	0.121
Distribution of myocardial hypercontraction bands	0.33 ± 0.21	0.17 ± 0.17	0.83 ± 0.31	0.50 ± 0.22	0.246

p**: Kruskal Wallis testsignificance value p < 0.05.

*p < 0.05: When compared to group MIR.

&p < 0.05: When compared to group HRSS.

### 3.2 Biochemical results

**Fig. 4:**
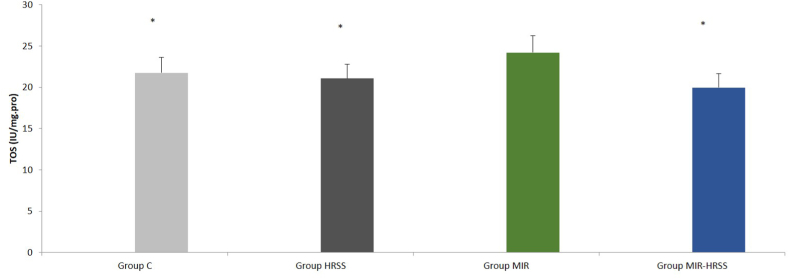
Myokardial tissue TOS levels. ∗*p* < 0*:*05: When compared to group MIR.

When the groups were compared among themselves in terms of TOS, there was a significant difference between the groups (p = 0.006). TOS was found to be significantly higher in the MIR group than in the C, HRSS and MIR-HRSS groups (p = 0.032, p = 0.008, p = 0.001, respectively), (Table 2, Fig. 4).

**Table 2:** Rat myocardial tissue oxidant status parameters [Mean ± SE].

	Group C (n = 6)	Group HRSS (n = 6)	Group MIR (n = 6)	Group MIR-HRSS (n = 6)	P**
TAS (nmol/mL)	1.60 ± 0.11*	1.49 ± 0.13*	1.32 ± 0.10	1.51 ± 0.10*	0.002
TOS (IU/mg.pro)	21.74 ± 1.86*	21.05 ± 1.75*	24.20 ± 2.05	19.97 ± 1.70*	0.006
ARES (IU/mg.pro)	353.17 ± 35.04	369.67 ± 7.86	365.50 ± 2.50	364.33 ± 4.93	0.922
PON (IU/mg.pro)	13.17 ± 2.75*	15.17 ± 2.59*	23.67 ± 2.56	15.17 ± 3.13*	0.036

p**: Kruskal Wallis test significance value p < 0.05.

*p < 0.05: When compared to group MIR.

**Fig. 5:**
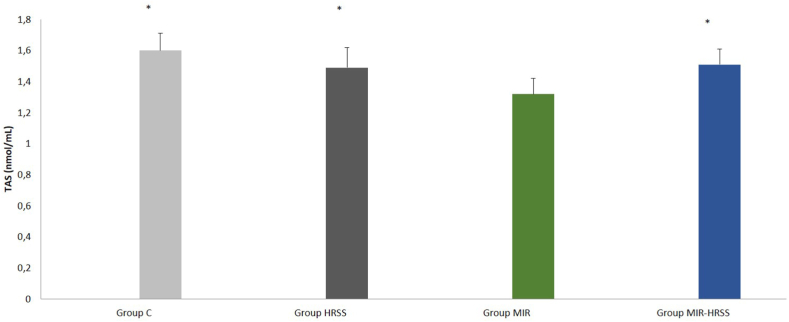
Myokardial tissue TAS levels. ∗*p* < 0*:*05: When compared to group MIR.

When the groups were compared among themselves in terms of TAS, there was a significant difference between the groups (p = 0.002). TAS was found to be significantly lower in the MIR group compared to the C, HRSS and MIR-HRSS groups (p < 0.0001, p = 0.016, p = 0.006, respectively), (Table 2, Fig. 5).

**Fig. 6:**
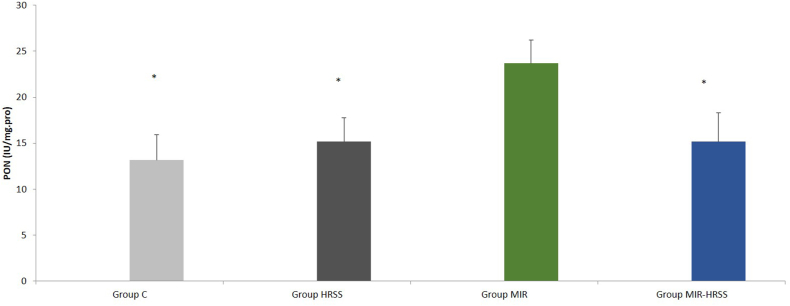
Myokardial tissue PON activity. ∗*p* < 0*:*05: When compared to group MIR.

Similarly, when the groups were compared among themselves in terms of PON enzyme activity, there was a significant difference between the groups (p = 0.036). PON enzyme activity was found to be significantly lower in the MIR group compared to the C, HRSS and MIR-HRSS groups (p = 0.014, p = 0.042 p = 0.042, respectively), (Table 2, Fig. 6).

**Fig. 7:**
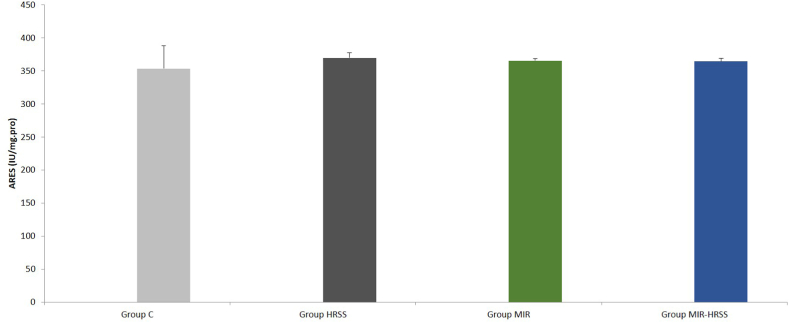
Myokardial tissue ARES activity.

Arylesterase enzyme activity was similar between the groups (p = 0.922), (Table 2, Fig. 7).

## 4 Discussion

This study demonstrates that HRSS has protective effects on MIR damage. In our research, we observed that the H2 molecule protects free oxygen radicals and exhibits antioxidant activity. With this effect, we observed that pre-reperfusion HRSS application provided significant histopathological improvement in the extent and severity of interstitial edema and cardiomyocyte degeneration in rats with MIR damage.

Ischemia/reperfusion injury is one of the most important factors affecting prognosis, surgical success and survival of patients. It is extremely important to develop new drugs and techniques to reduce the negative consequences of this damage. Today, iatrogenic myocardial ischemia followed by reperfusion with the use of heart/lung pumps in the procedures applied in cardiac surgery has caused studies on this subject to focus more on the heart. Although there are very important developments in the field of myocardial protection, the ideal drug, technique, solution or method to be used during reperfusion has not yet been clearly defined. This is explained by the inability to fully explain the mechanism of IR injury and the complexity in its physiopathology. The main focus of the studies on the subject is the inhibition of the production of free oxygen radicals in the pathogenesis of cell damage, the determination of the role of antioxidant mechanisms, and the trials of antioxidant free radical scavengers [16].

ROS is produced in the reperfusion of the ischemic myocardium and is considered an important cause of fatal reperfusion injury [17,18]. Radical oxygen species O_2_ and H_2_O_2_ are detoxified by antioxidant defense enzymes, unlike OH and ONOO, which no enzyme has been able to detoxify until now. Recently, hydrogen gas has been shown to selectively reduce these two harmful ROS [9]. Since the hydrogen molecule is electrically neutral and much smaller than the oxygen molecule, it easily penetrates membranes and enters cells and organelles such as nuclei and mitochondria. This is particularly important because the latter is the primary production site of ROS after reperfusion and is notoriously difficult to target [19]. Since the heart is one of the most highly perfused tissues, it is possible for the intramyocardial H_2_ concentration to increase immediately after intraperitoneal injection of hydrogen-rich saline.

In our study, when the MIR group and MIR-HRSS were compared, a significant difference was found between the oxidant status parameters.

In the treatment of heart diseases on the basis of ROS, studies using nanoencapsulation method are carried out in order to facilitate the entry of antioxidant substances into the cell [20]. In this study, we wanted to examine the effect of HRSS on MIR, which has few studies in the literature. We aim to apply the antioxidant substance that we will use in our future studies with nanoencapsulation.

There are studies showing that iv administration of hydrogen-rich saline during cardiopulmonary bypass in rats reduces myocardial damage [21]. In a study that examined the cardioprotective effect of hydrogen-rich saline at different doses [22] 5; 7.5; A cardioprotective effect was observed at all doses of 10 ml/kg HRSS. It was observed that the effect increased with increasing dose. In our study, HRSS was used as 10 ml/kg.

In our research, when the MIR group and MIR-HRSS were compared, a significant difference was found between the oxidant status parameters.

In our study, we obtained information about MIR in heart tissue by measuring PON and arylesterase. PON enzyme shows antioxidant activity by protecting LDL from oxidation and neutralizing free radicals including hydrogen peroxide [[23], [24], [25]]. When this balance is disturbed, oxidative damage occurs. Increased lipid peroxidation is also one of the consequences of increased oxidative stress [26]. In in vivo and in vitro studies, it has been reported that some parameters of erythrocyte functions and membrane integrity are impaired in increased lipid peroxidation. The resulting products due to lipid peroxidation associated with increased oxidative stress significantly affect membrane permeability and microviscosity. Thus, it was observed that the deformation capacity of erythrocytes decreased and they survived [27,28]. The decrease in PON and arylesterase enzyme activities in our study can be explained by the hypothesis of Cízová et al. that ischemia of the rat breed (depending on the ischemia duration) increases the natural antioxidant activity. In the study of Yazıcı et al. an increase in PON enzyme activity was observed in rats with intestinal IR damage during prolonged ischemia. However, further studies are needed to explain these data [29,30].

This study mainly focused on the beneficial effects of H_2_ saline on IR injury. However, its effect on permanent myocardial infarction, which has the same importance, has not been determined. Theoretically, it may be difficult for a single dose of H_2_ saline to induce potent cardioprotection in non-reperfused animals that develop myocardial infarction. Since permanent myocardial infarction is a long course and requires additional time to heal, H_2_ saline will only provide protection when a stable H_2_ concentration in the blood is maintained. This study does not include animals sacrificed at different periods following the H_2_ saline strain, which needs further research. Many studies [[31], [32], [33]] have been conducted on humans and rats regarding the genetic diversity in inflammatory and anti-inflammatory pathways effective in the pathogenesis of MIR injury. In our study, these genetic risk factors were neglected. This is among the limitations of our study. In addition, hemodynamic studies in H_2_ saline cells should be better after IR injuries, and only male rats were used as subjects, which are our study limitations. We aim to conduct studies using female rats and examining the long-term effects of HRSS.

## 5 Conclusion

In summary, our findings in this study show that hydrogen-rich saline solution has protective effects on IR injury. When HRSS was applied 5 min before reperfusion, it was observed that the severity of interstitial edema decreased, subendocardial haemorrhage decreased, and while it decreased the oxidant status parameters, it increased the antioxidant status parameters. We believe that when supported by other studies, the protective effects of HRSS on IR injury will be shown in detail and the indications for use will be expanded.

## Declaration of competing interest

The authors declare that they have no known competing financial interests or personal relationships that could have appeared to influence the work reported in this paper.
